# KaSARi: a national framework for standardized, automated, and predictive radiotherapy in Finland

**DOI:** 10.2340/1651-226X.2026.45682

**Published:** 2026-05-18

**Authors:** Jan Seppälä, Maria Tengström, Henri Korkalainen, Tuomas Virén, Juuso T.J. Honkanen, Juha Nikkinen, Kaisa Lehtiö, Annika Ålgars, Jani Keyriläinen, Sami Suilamo, Heidi Nurmi, Tanja Skyttä, Eeva Boman, Tero Vatanen, Liisa Sailas, Ulla-Mari Arkko, Kristiina Vuolukka, Tuomas Koivumäki, Tiina Metsäharju, Timo Voivalin, Janne Heikkilä

**Affiliations:** a Department of Radiotherapy, Kuopio University Hospital, Kuopio, Finland; b Department of Technical Physics, University of Eastern Finland, Kuopio, Finland; c Department of Radiotherapy, Research Unit of Health Sciences and Technology, Oulu University Hospital, University of Oulu, Oulu, Finland; d Department of Oncology and Radiotherapy, Turku University Hospital and University of Turku, Turku, Finland; e Department of Oncology, Tampere University Hospital, Wellbeing Services County of Pirkanmaa, Tampere, Finland; f Department of Clinical Physiology, Nuclear Medicine and Medical Physics, Tampere University Hospital, Wellbeing Services County of Pirkanmaa,Tampere, Finland; g Cancer Centre, North Carelia Central Hospital, Joensuu, Finland; h Department of Radiotherapy and Oncology, Wellbeing Services County of Central Finland, Jyväskylä, Finland

**Keywords:** Radiotherapy, national data collection, artificial intelligence, toxicity, quality of life

## Abstract

**Background and purpose:**

National radiotherapy (RT) data infrastructures are emerging to support treatment quality assurance and outcome research. However, prospective nationwide integration of full DICOM-RT data with toxicity and patient-reported outcome measures (PROMs) remains uncommon. KaSARi (Key advances in Standardizing, Automating, and predicting Risks in RT) was established to create national research infrastructure for prospective RT data collection and integration in Finland.

**Materials and methods:**

KaSARi is a prospective, consent-based multicenter cohort infrastructure involving Finnish university and central hospitals. All adult patients receiving RT are eligible. DICOM-RT datasets from treatment planning and oncology information systems are linked to clinician-graded toxicity, PROMs and relevant clinical variables. The infrastructure builds on previously validated multi-institutional DICOM workflows and follows FAIR (Findable, Accessible, Interoperable, Reusable) data principles. Predefined work packages support data harmonization, scalable data processing and enable downstream applications such as AI-based segmentation, dose prediction, and outcome modeling.

**Results:**

Patient recruitment started in May 2025. By February 2026, 221 patients had been enrolled at the coordinating center, initially including breast cancer patients and subsequently expanding to all RT indications. Recruitment at a second center began in November 2025, with 37 patients enrolled. The infrastructure is projected to include approximately 29,000 patients over 15 years. Integration of dosimetric, clinical and PROM data is progressing through a phased national implementation as additional centers join the network.

**Interpretation:**

KaSARi represents a national infrastructure prospectively integrating full DICOM-RT datasets with clinician-graded toxicity and PROMs across all RT indications within a unified research protocol. The study provides a foundation for nationwide outcome modeling, harmonization of RT practices and development and validation of data-driven AI-based methods.

## Introduction

Radiotherapy (RT) is one of the most data-intensive modalities in oncology, generating high-resolution image data, detailed structure delineations, 3D-dose distributions and RT plans for every treated patient. In principle, these data provide a rich source of information for evaluating treatment quality, studying dose–response relationships, and supporting the development of improved treatment strategies. However, in routine clinical practice, the data is typically stored locally within institutional systems and rarely centralized or systematically combined across treatment centers to facilitate large-scale research, benchmarking, or standardization of clinical practices. Conse-quently, although RT continues to deliver excellent treatment outcomes for many cancer types, the systematic investigation of outcomes in the era of modern RT remains relatively limited. The linkage between delivered dose, clinical decision-making and individual patient outcomes is therefore inconsistently implemented and remains incomplete [1].

Three structural limitations hinder data-driven optimization of RT. Firstly, target delineation remains variable across clinicians and institutions and the prospective linkage between contouring practices and clinical outcomes remains absent in routine clinical practice [2, 3]. Secondly, standardized dose prescriptions do not ensure uniform dose distributions. Substantial inter- and intra-institutional variability in target coverage and organ-at-risk exposure has been consistently demonstrated, even when identical clinical prescriptions are applied [4, 5]. Thirdly, reliable individual-level prediction of toxicity, recurrence and survival remains limited. Current predictive models rarely integrate voxel-level dose data, systemic therapy exposure, comorbidities and patient-reported outcome measures (PROMs) within a unified framework [6, 7].

In Denmark and Sweden, national RT infrastructures (DcmCollab and SKvaRT) have addressed important aspects of RT data harmonization [8, 9]. However, Finland has lacked a prospective framework integrating complete DICOM-RT datasets with structured outcome collection. RT data in Finland have historically been stored locally within institutional firewalls, with limited cross-center integration due to technical heterogeneity, governance structures, and data protection constraints. This decentralized landscape has restricted the development of large-scale datasets necessary to support systematic multi-center research and infrastructure-level standardization.

KaSARi (Key advances in Standardizing, Automating, and predicting Risks in RT) was established to fill this gap as a prospective, consent-based national RT research infrastructure. The primary aim of KaSARi is to design, implement and deploy a standardized framework for prospective, multicenter RT data integration in Finland. Rather than functioning solely as a registry or a single outcomes study, KaSARi is structured as a research protocol–driven platform that integrates comprehensive DICOM-RT datasets with structured clinical data, clinician-graded toxicity, and PROMs collected during routine care from patients treated with modern RT techniques. The infrastructure enables systematic aggregation of imaging, treatment planning, dose, and outcome data within a harmonized framework and supports scalable multicenter analyses. This platform further enables downstream applications, including outcome modeling and AI-based method development, as use-cases of the infrastructure rather than primary endpoints of this work. To our knowledge, no previous national initiative has prospectively integrated full DICOM-RT datasets, clinician-graded toxicity and PROMs for all RT indications under a unified study protocol.

## Material and methods

In Finland, RT is delivered in 12 cancer centers distributed nationwide, within five university hospital catchment areas. National coordination, harmonization and strategic development of cancer care are conducted through the Finnish Cancer Center (FICAN) network [10]. The Finnish Cancer Registry (FCR), established in 1952, provides population-based incidence and mortality data with national coverage [11, 12]. However, RT-specific information within the FCR and disease-specific registries remains limited and typically includes only treatment intent, start and end dates, and the total prescribed dose. Detailed dose distributions, structure sets and reported toxicity or PROMs are not systematically integrated at the national level.

KaSARi was established to address these limitations by creating a harmonized national infrastructure for standardized RT data integration and research utilization. KaSARi is a prospective, multicenter cohort infrastructure designed to collect standardized RT data from all consenting patients treated with RT at participating Finnish centers. The study functions as a long-term national research infrastructure rather than a single clinical trial, enabling multiple sub-studies within defined research work packages. This manuscript focuses on the design, implementation and early-phase deployment of the infrastructure, rather than reporting clinical or predictive outcomes.

All adult patients receiving RT at participating centers are eligible for inclusion upon provision of written informed consent. No diagnosis-specific exclusion criteria are applied. With incremental inclusion of centers, approximately 29,000 patients are projected to be enrolled over 15 years, reflecting nearly all RT-treated diseases in Finland.

## Data collection and integration framework

**Figure 1:**
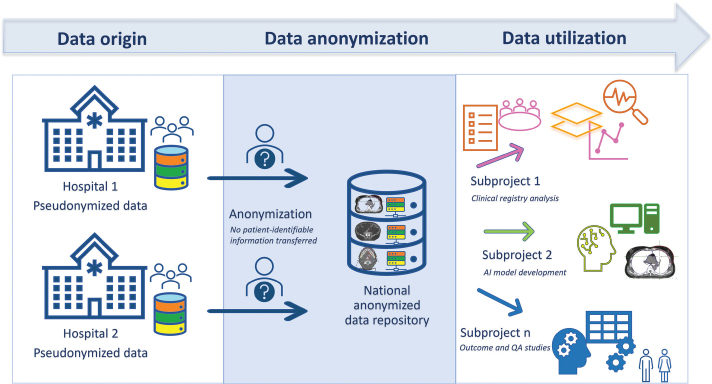
KaSARi data flow from hospital-level pseudonymization to national anonymized repository and research use. QA: quality assurance.

Original DICOM-RT datasets (RT Dose, RT Structure Set, RT Plan and treatment delivery summaries) are extracted from local treatment planning system (TPS)/oncology information system (OIS) environments. Data extraction is performed within institutional secure networks in compliance with Finnish and EU data protection regulations. Once the data are extracted, it is also pseudonymized (Figure 1). Structure nomenclature is harmonized using a standardized naming convention aligned with internationally recognized standards (AAPM TG-263) [13]. In cases where local naming deviates from the standard, structure labels are mapped to a unified nomenclature using predefined translation rules. Automated and semi-automated quality control procedures are implemented to detect inconsistencies in structure definitions, missing, or incomplete data.

The technical foundation of KaSARi builds upon earlier national development work. In 2019, an automated multi-institutional target delineation workflow was implemented across Finnish RT centers and presented at ESTRO 2019 [14]. That system included secure pseudonymization gateways, DICOM transfer via integration platforms, and centralized autosegmentation services operating within hospital firewalls. The successful implementation of this cross-center workflow demonstrated the feasibility of secure DICOM handling and multi-institutional data exchange within the Finnish RT environment. The architectural principles and secure data-transfer mechanisms developed in that project form part of the technical backbone of the current KaSARi infrastructure.

## Objectives

**Figure 2:**
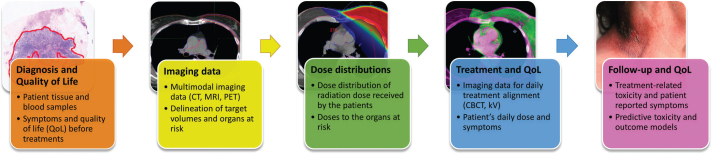
Multimodal data framework of KaSARi, linking baseline clinical variables, imaging and dose data, treatment monitoring, and longitudinal toxicity and quality of life (QoL) outcomes for predictive modeling. CT: computed tomography; MRI: magnetic resonance imaging; PET: positron emission tomography; CBCT: cone-beam computed tomography; kV: kilovolt imaging.

The primary objective of KaSARi is to establish a nationwide, standardized research infrastructure and repository for the prospective collection and integration of RT imaging, 3D-dose distributions, and clinical outcomes across participating centers (Figure 2). By establishing this unified framework, the infrastructure aims to facilitate large-scale evaluation of treatment delivery, outcomes, and practice patterns in modern RT.

In addition to establishing the infrastructure, KaSARi entails secondary research applications enabled by the platform. These are divided into three research objectives:

- To develop and validate AI-based segmentation and treatment planning tools (i.e. dose prediction and automated planning) using real-world clinical datasets.
- To model treatment-related toxicity and quality of life (QoL) outcomes to support data-driven normal tissue complication probability (NTCP) prediction, while accounting for the interactions between RT, systemic therapies, comorbidities, and baseline patient-reported health status.
- To facilitate federated learning and structured collaboration both nationally and across Nordic RT infrastructures.

## Participating centers and governance

The consortium currently includes, Kuopio University Hospital (KUH, coordinating center), North Karelia Central Hospital (PKKS), Oulu University Hospital (OYS), Tampere University Hospital (Tays), Turku University Hospital (Tyks) and University of Eastern Finland (UEF, data science partner). KaSARi operates under a national consortium agreement that defines the common governance, data-sharing principles, and operational framework of the infrastructure. All 12 Finnish municipal RT centers are eligible to join through formal accession to this agreement. This structured yet open model supports nationwide representativeness across diverse RT platforms, clinical workflows and patient populations.

## Results

The research protocol received ethical approval in April 2024 from the Medical Research Ethics Committee of the Wellbeing Services County of North Savo (23/2024). The approval covers the multicenter study framework and allows participating centers to join the study through the national consortium agreement. The following results describe the implementation status and early deployment of the infrastructure, rather than clinical or predictive outcomes.

Patient recruitment started at KUH on May 13, 2025, initially including breast cancer patients only. By the end of 2025, 106 of 206 eligible breast cancer patients had been enrolled, corresponding to a recruitment rate of 51%, demonstrating feasibility of prospective consent-based recruitment within routine clinical workflows, with stable recruitment rates observed during early scaling. Among enrolled patients, 59% completed electronic PROM questionnaires, reflecting early-stage PROM integration within the infrastructure. At the beginning of 2026, recruitment was expanded to include all RT patients at the coordinating center. By the end of February 2026, a total of 221 patients had been enrolled. During the subsequent period from February to March 2026, recruitment continued at a comparable level, with 111 patients enrolled out of 238 RT-treated patients at the coordinating center, corresponding to a recruitment rate of approximately 47% during this early expansion phase. The diagnostic distribution reflects routine clinical practice and includes breast, prostate, lung, and other RT-treated diseases.

Recruitment at Tyks began on November 2025, initially focusing on breast cancer patients. Since initiation, 37 patients have been enrolled, representing approximately 25% of eligible breast cancer patients during the recruitment period. Expansion to additional treatment sites at Tyks is planned. Progressive inclusion of additional Finnish RT centers is ongoing.

Implementation has proceeded in phases to establish recruitment pathways and demonstrate the feasibility of centralized data collection prior to nationwide expansion, reflecting the staged deployment of the infrastructure rather than finalized system maturity. Local data intake is fully operational at the coordinating center, while data intake procedures are being established at participating hospitals. Two additional Finnish RT centers are currently in the process of joining the consortium, and patient recruitment is scheduled to begin shortly in two further hospitals already involved in the network.

The data pipeline has now been successfully established, confirming technical feasibility of standardized multicenter data integration. DICOM-RT datasets are exported from the TPS and OIS to a pseudonymized local intermediate database. Following local validation, the datasets are anonymized and transferred to the national KaSARi repository hosted within a controlled-access research environment at KUH (Figure 1). Metadata, clinical toxicity data, and PROMs are linked via identifiers, enabling longitudinal follow-up across the treatment trajectory, with harmonization procedures applied to ensure consistency of structure definitions, toxicity grading, and PROM datasets across centers.

PROM collection has been successfully integrated at KUH and will be expanded to other participating centers, with current participation rates reflecting early-phase implementation and are expected to improve with workflow standardization and broader system integration. PROMs are collected digitally whenever feasible using validated standardized instruments, including the European Organisation for Research and Treatment of Cancer (EORTC) QLQ-C30 [15]. Questionnaires are administered at predefined time points before, during, and after treatment, enabling temporal correlation between delivered dose metrics and patient-reported toxicity. Information on systemic medication, comorbidities, blood sample data, tissue samples, and physician-assessed toxicities is recorded within the OIS and linked to the corresponding DICOM-RT datasets.

Each complete RT dataset, including imaging, dose distributions, and structure sets, averages approximately 0.5–1 GB per patient. With progressive national expansion, annual data growth is projected to reach several terabytes. Scalable and secure storage is implemented within the Finnish national research data infrastructure, ensuring compliance with data protection regulations while enabling controlled research access.

## Discussion and conclusion

Despite the increasing availability of detailed digital RT data, systematic infrastructures linking treatment delivery, dose distributions, and clinical outcomes remain limited in many healthcare systems. In particular, large-scale integration of 3D-dose information, structure delineations, and longitudinal PROMs has been inconsistently implemented, restricting opportunities for comprehensive evaluation of treatment practices and outcomes in modern RT. KaSARi was established to address this gap through a national, prospective collection of DICOM-RT data linked with clinician-graded toxicity and PROMs under a unified national governance framework within Finland. Unlike registry-driven infrastructures, KaSARi operates as a research protocol-based architecture, enabling prospective harmonization of technical dose data and clinical outcomes. This work describes the infrastructure and its early implementation, rather than reporting outcome analyses derived from the dataset.

A major strength of KaSARi is its prospective design. Data are collected under a standardized informed consent and harmonized nationally. This prospective approach allows consistent data collection, thereby reducing the risk of missing or incomplete data that often limits retrospective analyses. In addition, prospective governance enables continuous refinement of data collection procedures, allowing new variables, outcome measures, or technical parameters to be incorporated in a coordinated manner across centers as clinical practice evolves. This flexibility facilitates the systematic harmonization of datasets and ensures that the infrastructure remains aligned with emerging research needs and technological developments.

Another major strength of KaSARi is the integration of technical RT data with both clinician-assessed toxicity and PROMs, although current PROM completeness and consent-based inclusion introduce potential limitations in represen-tativeness that require consideration in downstream analyses. By linking 3D-dose distributions and structure data with longitudinal clinical and patient-reported outcomes, the platform enables detailed investigation of dose–response relationships and supports development of NTCP models that incorporate both clinical toxicity grading and patient-centered outcomes. Finally, methodological development, including AI-based approaches, is embedded within the infrastructure and is presented here as an enabled application of the platform rather than a primary outcome of this study. Activities such as validation of autosegmentation methods, development of dose prediction and automated planning approaches, and dose-based predictive modeling are incorporated as predefined research work rather than retrospective secondary analyses. Embedding these activities within the prospective data collection framework enables systematic evaluation of new methods using harmonized multicenter datasets while maintaining close integration with routine clinical workflows.

The consent-based design and incomplete PROM participation introduce potential selection bias that should be considered in future analyses. Patients providing informed consent may differ systematically from non-participants (age, performance status, comorbidity burden), while PROM completion may depend on symptom severity, patient engagement, or technical accessibility, leading to non-random missingness. These factors may affect representativeness and bias downstream analyses, particularly in predictive modeling and AI development.

The observed recruitment rate (~50%) and PROM completion rate (~60%) reflect an early implementation phase of the infrastructure. Contributing factors include variability in the integration of consent procedures into clinical workflows, patient-related characteristics, and logistical aspects related to the introduction of electronic PROM systems and staff familiarity with study processes. These factors are largely modifiable. Ongoing efforts focus on embedding consent procedures into routine clinical pathways, improving usability and accessibility of digital PROM platforms, and increasing staff training and engagement across centers. The prospective design of KaSARi enables continuous monitoring and iterative optimization of recruitment and data collection processes. Accordingly, future analyses will incorporate appropriate missing data handling and bias-adjustment methods, while participation rates and data completeness are expected to improve as workflows become more standardized and integrated into routine practice.

Several Nordic initiatives have pioneered national RT data harmonization. Recent national audit data from Denmark demonstrate both the increasing complexity of RT practice and substantial variability in treatment indications and dose prescriptions, underscoring the need for standardized, prospective data infrastructures [16]. However, their conceptual frameworks differ from that of KaSARi (Table 1). DcmCollab in Denmark provides storage of raw DICOM datasets primarily derived from clinical trial cohorts. The platform can support integration with toxicity grading and PROM data, particularly within structured study settings. This approach offers maximal flexibility for retrospective dosimetric analyses but is typically based on study-specific or optional data linkage rather than a uniformly applied prospective framework. SKvaRT in Sweden aggregates condensed RT variables for all Swedish patients into a national quality registry. The primary focus is quality assurance and clinical benchmarking and data are intentionally limited to predefined dose-volume descriptors rather than full DICOM datasets. KaSARi complements these models by prospectively integrating complete DICOM-RT datasets with clinician-graded toxicity and longitudinal PROMs within an infrastructure framework designed to support, rather than directly report, downstream analytical and predictive studies. In this sense, KaSARi combines the research-oriented granularity of DcmCollab with the nationwide coverage ambition of SKvaRT, while embedding AI development and predictive modeling as explicit infrastructure objectives. The infrastructure is further designed to support direct use of harmonized multimodal data in predictive modeling and AI development without requiring additional study-specific data integration steps.

**Table 1:** Comparative overview of the Finnish (KaSARi), Danish (DcmCollab) and Swedish (SKvaRT) national RT data infrastructures.

Feature	KaSARi	DcmCollab	SKvaRT
Data detail	Full DICOM-RT + PROMs	Full raw DICOM-RT	Condensed RT variables
Collection type	Prospective research protocol	Retrospective trial-linked	Automated registry extraction
Outcome linkage	PROMs, survival, CTCAE, biomarkers	Trial-specific endpoints	Survival & limited toxicity
Primary goal	AI & individual risk modeling	Data fidelity & trial storage	Clinical quality monitoring

PROMs: patient-reported outcome measures; CTCAE: Common Terminology Criteria for Adverse Events.

Early implementation has highlighted several technical and organizational challenges. Foremost among these is the need for sustainable long-term funding for both research activities and information technology infrastructure. The development and maintenance of secure data storage solutions and robust data pipelines require operational continuity that extends beyond traditional project-based funding models. Secondly, national consensus on structure naming conventions and data dictionaries is critical. As observed in other harmonization efforts, target volumes are more challenging to standardize than organs at risk. While KaSARi adopts internationally recognized nomenclature standards (AAPM TG-263) comple-mented by ESTRO contouring guidelines, there is ongoing national coordination to maintain semantic consistency as clinical practices evolve, including iterative updates to resolve differences in structure definitions across centers.

A specific and important challenge concerns harmonization of toxicity grading and PROM collection across centers. Although KaSARi aims to implement common terminology criteria for adverse events (CTCAE) as the uniform framework for clinician-graded toxicity, local practices vary. To address this, shared national guidelines and training will be implemented to support consistent interpretation and application of CTCAE across centers. KUH utilizes the Kaiku Health (Elekta, Stockholm, Sweden) platform for PROM collection [17], whereas other centers employ locally developed or other electronic health record systems. To reduce heterogeneity, KaSARi prioritizes the use of standardized PROM instruments (e.g. EORTC QLQ-C30) and aims to align data collection timepoints across centers to ensure comparability of outcomes. This heterogeneity can affect data comparability and complicates automated longitudinal analyses. Establishment of standardized electronic questionnaires and interoperable national interfaces would therefore be beneficial to ensure consistent PROM acquisition and seamless database integration. However, the active multicenter collaboration within the project is expected to harmonize the clinical practices and enable consistent data collection even with differing PROM acquisitions. Importantly, harmonization within KaSARi is implemented as an iterative and continuous process rather than a one-time standardization step, with prospective data collection enabling ongoing refinement, validation and feedback across participating centers.

A critical requirement for the long-term sustainability of KaSARi is the establishment of stable funding models that extend beyond traditional project-based research grants. The development and maintenance of national RT data infrastructures involve continuous costs related to data storage, secure computing environments, system maintenance and dedicated personnel. Currently, KaSARi operates through a combination of research funding and institutional support. However, long-term sustainability will likely require integration of infrastructure funding into national research infrastructure frameworks and healthcare system funding structures, supported by coordinated national-level strategies, for example, through the FICAN.

Although KaSARi is developed within the Finnish healthcare system, several core design principles support its transferability to other national contexts. The use of standardized data formats (DICOM-RT) and adherence to FAIR (Findable, Accessible, Interoperable, Reusable) data principles enable interoperability across different technical environments. The architecture, which combines local data storage with centralized or distributed analysis, allows flexibility in implementation depending on national data governance requirements. In addition, the consortium-based model provides a scalable governance structure that can be adapted to healthcare systems with varying degrees of centralization. However, successful implementation in other countries would depend on factors such as national data protection regulations and availability of digital infrastructure. Federated learning approaches may further facilitate international collaboration by enabling model development and validation across institutions without the need to transfer patient-level data.

In the coming years, KaSARi is expected to expand to the majority of Finnish RT centers with the current work representing an initial phase in establishing a scalable national infrastructure. The consortium-based model enables every municipal RT unit to join under a unified governance and technical framework, ensuring nationwide representativeness while maintaining standardized data acquisition. A key priority is the establishment of a permanent national structure to support harmonization of RT practices. In parallel, dashboard-based feedback tools will be implemented to enable real-time benchmarking and support continuous clinical quality improvement across centers. Further development includes the implementation of federated learning pipelines in collaboration with Nordic partners, enabling the secure exchange and validation of AI models without the transfer of raw patient-level data. This approach supports scalable multicenter model development while preserving data protection and institutional autonomy.

KaSARi will also align with international initiatives on RT data standardization and FAIR data principles to ensure interoperability, long-term sustainability, and research reproducibility. As data standards in radiation oncology continue to evolve, the integration of KaSARi variables with structured and harmonized data models will further strengthen consistency, and enable further national and international collaboration.

## Conclusions

KaSARi is establishing Finland’s first prospective national RT cohort combining complete DICOM-RT datasets with clinician-graded toxicity and PROM values under a unified research model. This manuscript describes the design and early deployment of the infrastructure, which is intended to support future outcome analyses and predictive modeling. By embedding prospective data collection into routine clinical workflow, KaSARi provides a sustainable foundation for nationwide outcome evaluation, AI-driven treatment optimization, and predictive modeling of radiation-induced toxicity. The infrastructure enables the harmonization of RT practices throughout Finland while maintaining the detailed dose data needed for high-resolution research.

Prospective integration of technical RT data with longitudinal clinical outcomes is expected to improve the comparability of treatments, strengthen the development of predictive models and ultimately support more individualized RT with reduced toxicity and improved patient QoL.

## Data Availability

The datasets generated are part of the KaSARi national radiotherapy research infrastructure and are not publicly available. Access to the data is restricted to participating centers under the KaSARi governance framework and in accordance with informed consent provided by the patients and applicable data protection legislation.
